# Ultra-small-angle neutron scattering with azimuthal asymmetry

**DOI:** 10.1107/S1600576716005586

**Published:** 2016-05-16

**Authors:** X. Gu, D. F. R. Mildner

**Affiliations:** a Department of Geosciences, Pennsylvania State University, University Park, PA 16802, USA; b NIST Center for Neutron Research, National Institute of Standards and Technology, Gaithersburg, MD 20899, USA

**Keywords:** azimuthal asymmetric scattering, double-crystal diffractometers, elliptic scattering contours, slit-height smeared scattering, small-angle neutron scattering

## Abstract

Small-angle neutron scattering (SANS) measurements from thin sections of rock samples such as shales demand as great a scattering vector range as possible because the pores cover a wide range of sizes. The limitation of the scattering vector range for pinhole SANS requires slit-smeared ultra-SANS (USANS) measurements that need to be converted to pinhole geometry. The desmearing algorithm is only successful for azimuthally symmetric data. Scattering from samples cut parallel to the plane of bedding is symmetric, exhibiting circular contours on a two-dimensional detector. Samples cut perpendicular to the bedding show elliptically dependent contours with the long axis corresponding to the normal to the bedding plane. A method is given for converting such asymmetric data collected on a double-crystal diffractometer for concatenation with the usual pinhole-geometry SANS data. The aspect ratio from the SANS data is used to modify the slit-smeared USANS data to produce quasi-symmetric contours. Rotation of the sample about the incident beam may result in symmetric data but cannot extract the same information as obtained from pinhole geometry.

## Abstract 2

A method is given for converting slit-smeared azimuthally asymmetric data collected on a double-crystal diffractometer for concatenation with the usual pinhole-geometry small-angle neuton scattering data.

## 1. Introduction   

The typical range of scattering vectors measured on a pinhole small-angle neutron scattering (SANS) instrument is 10^−3^ < *Q* < 0.5 Å^−1^, suitable for measuring correlations and dimensions in the range 10–1000 Å. The modulus of the scattering vector is defined by *Q* = (4π/λ)sin(θ/2), or *Q* ≃ 2πθ/λ at small angles, where λ is the wavelength and θ is the scattering angle, and the measured dimensions are in the range of ∼*Q* ^−1^. The circular isointensity contours on an area detector for isotropic systems that scatter with azimuthal symmetry may be azimuthally averaged to obtain a one-dimensional plot of the scattered intensity *I*(*Q*) as a function of *Q*. Moreover, a two-dimensional detector enables observation of the intensity from azimuthally asymmetric scattering samples. Efforts are underway to extend the lower end of the *Q* range by a further order of magnitude using pinhole geometry, in order to examine dimensions as large as 1 µm (Brûlet *et al.*, 2008 ▸; Dewhurst, 2014 ▸; Barker, 2015 ▸; Abbas *et al.*, 2015 ▸).

For systems having dimensions greater than 1 µm, an alternative method is required to extend the measurable range to lower values of the scattering vector. Ultra-small-angle neutron scattering (USANS) using a double-perfect-crystal diffractometer can measure in the scattering vector range of 10^−5^ < *Q* < 10^−3^ Å^−1^ and examine dimensions in the range 0.1–10 µm (Agamalian *et al.*, 1997 ▸; Barker *et al.*, 2005 ▸). This instrumental arrangement is different in concept from pinhole SANS in that it measures with high resolution in one direction only, while having a much broader angular acceptance in the orthogonal direction in order to increase the count rate. This has been made possible by improvements to the Bonse–Hart arrangement (Bonse & Hart, 1965 ▸) of silicon channel-cut crystals by the elimination of the wings on the rocking curve (Agamalian *et al.*, 1997 ▸).

The smearing of the scattered intensity for USANS is similar to slit-smeared small-angle X-ray scattering. If a model that describes the scattering system exists, the smeared scattering function should be compared with the USANS data. However, if such a model is unavailable, it is necessary to desmear the USANS data in order to place the scattered intensity *I*(*Q*) on an absolute basis with the SANS data from a conventional pinhole instrument. The desmeared USANS intensity from systems that scatter with azimuthal symmetry may conveniently be concatenated with the azimuthally averaged data obtained from the accompanying SANS measurement. This procedure is not available for anisotropic systems and consequently there are few USANS data analyzed on azimuthally asymmetric systems. We examine the analysis of systems for which the scattering pattern consists of elliptical isointensity contours.

Asymmetric data often have contours with an elliptical dependence, which indicate that the scattering is from entities that have azimuthal symmetry about some unique axis within the system (Summerfield & Mildner, 1983 ▸). Ciccariello *et al.* (2000 ▸) have generalized the Porod law as a function of the direction of *Q* for anisotropic samples. The unique axis may be a stretched direction as in extruded materials (such as the uniaxial drawing of polyethylene; Matsuba *et al.*, 2013 ▸), or it may be caused by uniaxial compaction as in geological samples (Gu *et al.*, 2015 ▸). Sector averages over a range of azimuthal angles are taken so that the scattered intensity *I*(*Q*) is obtained with reasonable statistics along the two orthogonal directions.

The desmearing of USANS data for conversion to pinhole geometry uses an iterative method (Lake, 1967 ▸) that is good for symmetric scattering data but not for asymmetric scattering because the two orthogonal dimensions are not commensurate. We investigate the information that might be gleaned from USANS data collected from such anisotropic systems by taking measurements from rock samples in different orientations.

## 2. Geological samples   

USANS measures the scattering of neutrons from scattering length density (SLD) fluctuations in real space in the micrometre range. It is particularly useful for systems in materials science and polymer science that are optically opaque and therefore cannot be examined by light scattering. The technique is also useful for the study of porous media such as cements. Here, we consider a geological example to probe the difficulties of treating asymmetric scattering systems. [For a review, see Radlinski (2006 ▸)]. We assume that we have a two-phase system with scattering from pores within the rock, arising from the difference in SLD of the pores relative to some average SLD of the rock matrix.

The sample used in this study is a Dunkirk/Huron black shale collected from an outcrop at Erie, Pennsylvania, USA. The Dunkirk/Huron black shale is an important Devonian-aged source rock and gas-producing reservoir in the Appalachian Basin (Milici & Swezey, 2006 ▸). The sample consists of 42.5 wt% quartz, 37 wt% illite, 8 wt% plagioclase, 6.5 wt% chlorite and 6 wt% pyrite (determined by X-ray diffraction). The total organic carbon (TOC) content is 8.33 wt% (determined by carbon combustion). The SLD of the rock matrix is approximately 3.9 × 10^−6^ Å^−2^, which is close to the typical SLD of black shale (Gu *et al.*, 2015 ▸, 2016 ▸) and much greater than that of the empty pores (for which the SLD ≃ 0). Differences in random composition within the rock lead to additional background in the scattering data.

The pores in rocks and shales form a fractal microstructure that gives rise to intense power-law scattering, so that there is always multiple scattering present for geological samples. Triolo *et al.* (2000 ▸) and Radlinski *et al.* (2000 ▸) have shown that multiple scattering affects this dependence, causing deviation from the power-law scattering resulting in incorrect analysis. Even 1 mm thick samples are subject to strong multiple scattering. SANS measurements can benefit from having a shorter wavelength, whereas the very small angles of USANS require geological samples about 150 µm thick (Anovitz *et al.*, 2009 ▸), a reasonable compromise between an adequate scattered signal and excessive multiple scattering.

**Figure 1:**
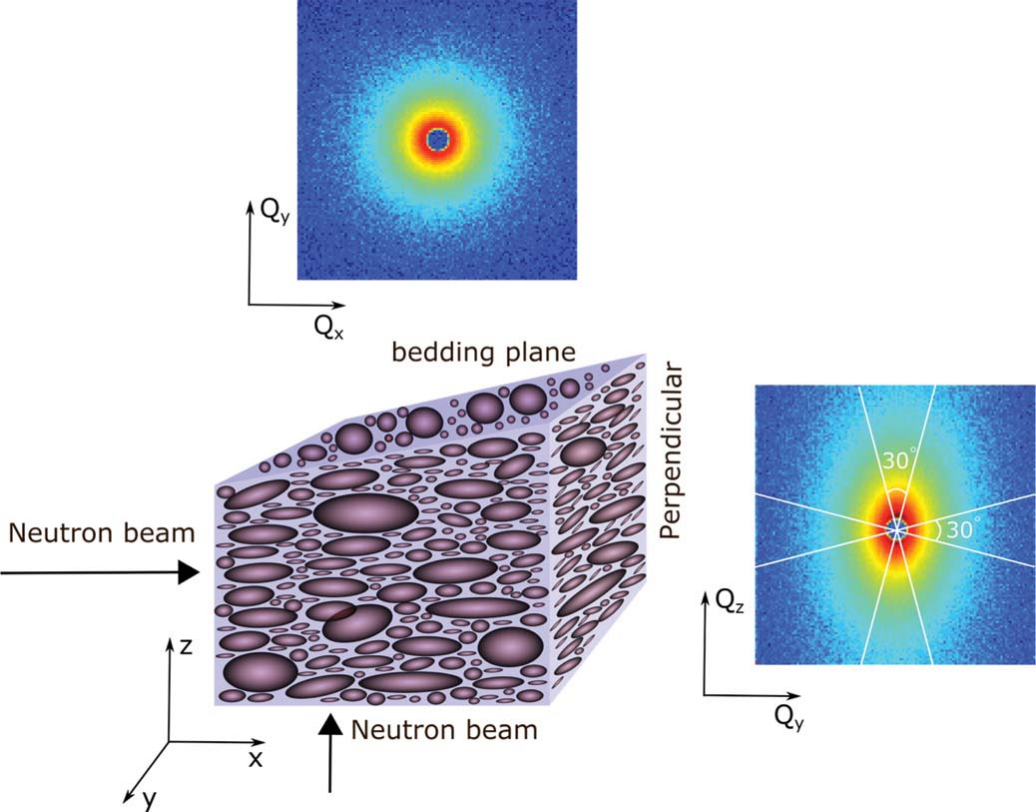
A schematic diagram indicating the planes of the two cuts, one parallel and the other perpendicular to the plane of bedding of the sample rock. The unique axis is the normal to the bedding plane. For each orientation of the sample, the scattered neutron beam results in a pattern on the two-dimensional detector. The scattering has circular (azimuthally symmetric) contours when the beam is incident along the *Z* direction on the sample cut parallel to the bedding plane *XY*, and elliptical (azimuthally asymmetric) contours when the beam is incident along the *X* direction on the sample cut perpendicular to the bedding plane *YZ*.

We compare measurements when the scattering plane is normal to the unique axis with those when the scattering plane includes the unique axis. In the case of geological samples, the unique axis is perpendicular to the plane of bedding. Measurements have therefore been taken on thin sections of rock that have been sectioned both parallel to and normal to the bedding plane (see Fig. 1 ▸). We assume that both cuts are representative of the particular rock system. Each sample was fixed onto a quartz glass slide of thickness 1 mm with negligible scattering, which was itself attached to a gadolinium aperture of diameter 12.7 or 6.35 mm, depending on the size of the sample. Though these sections had a nominal thickness of 150 µm, micrometer measurements indicated that the cut parallel to the bedding was 299 µm thick (within 10 µm), and the cut perpendicular to the bedding was 238 µm thick.

The SANS measurements were performed on the pinhole SANS instrument NG3 (Glinka *et al.*, 1998 ▸) at the NIST Center for Neutron Research (NCNR) in three different configurations, with Δλ/λ ≃ 12% and sample-to-detector distances of 1 and 4 m using 6 Å neutrons, and of 13 m. The last configuration used MgF_2_ lenses in order to extend the measurable range of the scattering vector (Choi *et al.*, 2000 ▸) and a wavelength λ = 8.09 Å, to enable *Q* = 1 × 10^−3^ Å^−1^ to be reached for an overlap with the USANS range. Measurements were taken on both the parallel- and perpendicular-cut samples, and also with the perpendicular-cut sample rotated continuously in the beam. Labeled in Fig. 1 ▸ are the three principal directions of the sample, *X* and *Y* within the bedding plane, and *Z* the normal to the plane. After background subtraction using a clean quartz slide measurement, the data for the parallel-cut sample were averaged azimuthally to produce *I*(*Q*). Sector averaging over azimuthal angles Δφ ≃ 30° is necessary for the perpendicular-cut measurement (averaging using Δφ ≃ 15° with fewer pixels results in noisier data).

Similar USANS measurements were performed on the double-crystal instrument BT5 (Barker *et al.*, 2005 ▸) at the NCNR using a wavelength λ = 2.38 Å with Δλ/λ = 5.9%. The double-crystal arrangement has an instrumental (full width at half-maximum) resolution of 2 × 10^−5^ Å^−1^ in the horizontal direction, with a *Q* range of 3 × 10^−5^ < *Q* < 3 × 10^−3^ Å^−1^, and with a relaxed resolution in the vertical *y* direction (± 0.117 Å^−1^). Note that the perpendicular-cut sample was first mounted with the bedding (direction *Y*) horizontal, in the direction of the high-resolution USANS scan, and then remounted so that the normal to the bedding (direction *Z*) was in the *Q* scan direction. Both sets of measurements were reduced to an absolute scale using the NIST data-reduction procedure (Kline, 2006 ▸).

## 3. SANS results   

Fig. 1 ▸ shows the two-dimensional SANS scattering patterns for the samples cut parallel and perpendicular to the bedding plane. We label the three principal directions, so that the bedding plane is always the *X* *Y* plane with the neutron beam in the *Z* direction, and the perpendicular-cut sample defines the *Y* *Z* plane with the neutron beam in the *X* direction. The two-dimensional SANS patterns are plots of *I*(*Q_x_*, *Q_y_*, 0) with the neutron beam along *Z*, and *I*(0, *Q_y_*, *Q_z_*) with the neutron beam along *X*.

Scattering from the perpendicular-cut sample results in elliptical isointensity contours. This signifies that the scattering is from entities that have azimuthal symmetry about some unique axis within the system. The assumption is that the rock is a two-phase scattering system, with pores that are flattened such that, on average, they are isotropic (randomly oriented) within the plane of the bedding, with the normal to the bedding as the unique axis. We also assume that the average pore dimension in this direction is less than that within the plane of bedding. Hence the scattering pattern for the perpendicular-cut sample has an elliptical form of isointensity contours, with the short axis corresponding to the *Y* direction of the bedding plane and the long axis corresponding to *Z*, the direction normal to the bedding.

**Figure 2:**
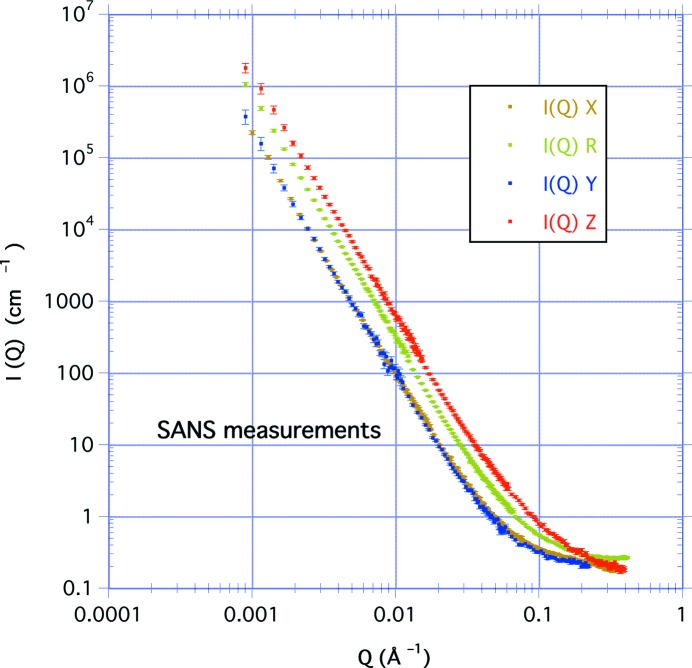
The azimuthally averaged SANS results, with intensity plotted as a function of *Q* over the range 0.001 < *Q* < 0.4 Å^−1^. The *X* curve is from the azimuthal average for the parallel-cut sample, the *Y* and *Z* curves are for sector averages in directions parallel and normal to the bedding for the perpendicular-cut sample, respectively, and the R curve is for the rotation of the perpendicular-cut sample. Note that the *Y* curve is coincident with the *X* curve since they are equivalent.

Fig. 2 ▸ shows the azimuthally averaged SANS results, indicating the intensity as a function of *Q* over the range 0.001 < *Q* < 0.4 Å^−1^ after correction for background and transmission. The measurement labeled *X* is the azimuthally averaged value of the symmetric scattering for the sample cut in a plane parallel to the bedding, *i.e.*

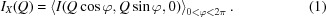
The perpendicular-cut measurement has elliptical isointensity contours, with *Y* and *Z* referring to the 30° sector average about the shorter axis and along the longer axis of the contours, respectively, *i.e.*

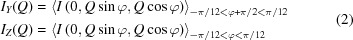
where φ is the azimuthal angle defined relative to the *Z* direction. Note that the short axis corresponds to the larger dimension in real space and is therefore the *Y* direction which is within the bedding plane. Consequently, the values for *Y* are coincident with those for *X*.

We can perform straight-line fits to the log–log plot, assuming that, over the range 0.001 < *Q* < 0.02 Å^−1^, the scattering intensity obeys a power law 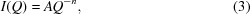
where *A* is a prefactor. Table 1 ▸ gives the results. We find that, over this *Q* range, all the measurements can be fitted with the same power-law exponent *n* = 3.33 (4).

We assume that the *Q* dependence of the scattering is separable from the azimuthal dependence, so that we may write the intensity as 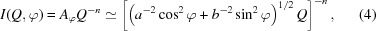
where φ is the azimuthal angle relative to the normal to the bedding plane. This approximation is reasonable provided that the *Q* dependence does not change over the *Q* range considered. This allows the possibility of elliptically averaging the data (Reynolds & Mildner, 1984 ▸).

**Figure 3:**
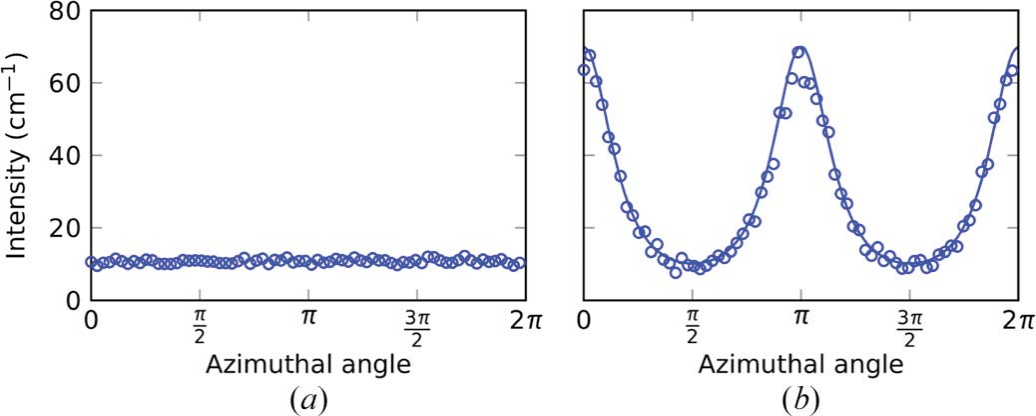
The scattered intensity at *Q* = 0.02 Å^−1^ as a function of azimuth when (*a*) the sample is cut parallel to the bedding with scattering in the *XY* plane, and (*b*) the sample is cut perpendicular to the bedding with scattering in the *YZ* plane. This shows that the *Y* measurement (φ = ±π/2) has a similar intensity to the *X* measurement. The *YZ* data are fitted to equation (4) so that *I*(φ) ≃ [cos^2^φ + (*a*/*b*)^2^sin^2^φ]^−*n*/2^, where *a*/*b* = 1.76 and *n* = 3.33.

**Figure 4:**
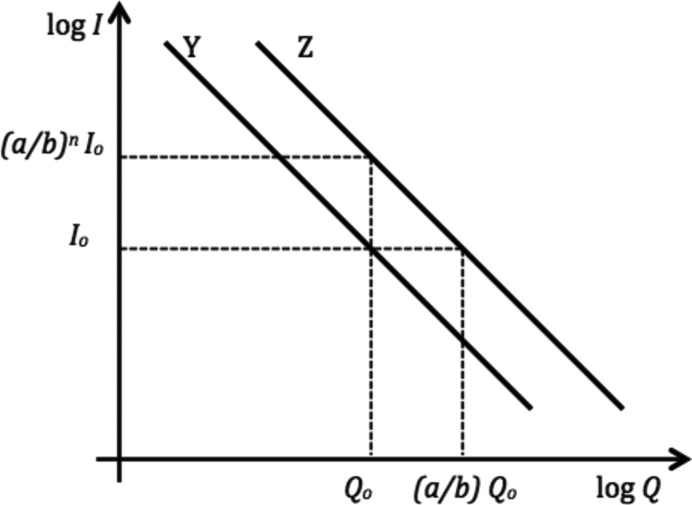
A schematic diagram showing intensity *I versus Q* for the *Y* and *Z* measurements, indicating that the two curves can be made coincident either (i) by multiplying the *Q* coordinate for the *Z* data by *b*/*a* or (ii) by multiplying the *Z* intensity values by (*b*/*a*)*^n^*.

Fig. 3 ▸ illustrates the anisotropy by showing the scattering intensity for the perpendicular-cut sample at *Q* = 0.02 Å^−1^ as a function of azimuthal angle. In the φ = 0 (or *Z*) direction, *I*_*Z*_(*Q*) = *A*_*Z*_ *Q* ^−*n*^ ≃ *a^n^Q* ^−*n*^, and in the φ = π/2 (or *Y*) direction, *I*_*Y*_(*Q*) = *A*_*Y*_ *Q* ^−*n*^ ≃ *b^n^Q* ^−*n*^. The factor *a*/*b* corresponds to the ratio of the long (φ = 0) and short (φ = π/2) semi-axes of the isointensity contours. Also, since *A*_*Z*_/*A*_*Y*_ = (*a*/*b*)^*n*^, shifting the values of the coordinate *Q* to (*b*/*a*)*Q* for the *Z* data allows all three measurements to be made coincident. Alternatively, Fig. 4 ▸ shows that the same result can be obtained by multiplying the intensity values for the *Z* plot by (*b*/*a*)*^n^*. (A similar relationship will be used in the analysis of the smeared USANS data.)

**Figure 5:**
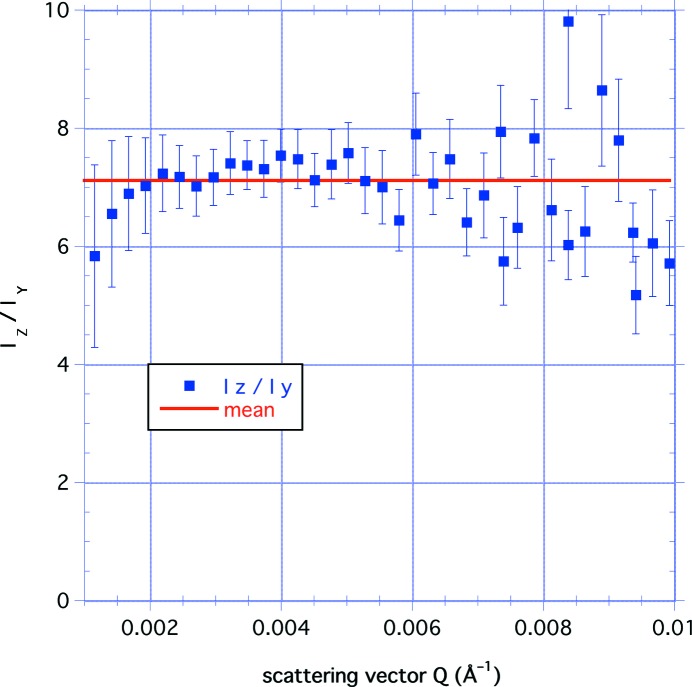
The ratio of the long- to short-axis intensities from the *Z* and *Y* curves, plotted as a function of *Q* for the range 1 × 10^−3^ < *Q* < 1 × 10^−2^ Å^−1^ with a mean value 7.11 (14). Taking the exponent *n* = 3.33 (4), this indicates a mean value for *a*/*b* = 1.80 (2).

From the fitted parameters given in Table 1 ▸, we find the value of *A*_*Z*_/*A*_*Y*_ = 6.3 (10) (all quoted uncertainties and error bars are one standard deviation). Assuming *n* = 3.33 (4), this indicates that the ratio *a*/*b* of the elliptical contours has a mean value for *a*/*b* = (*A*_*Z*_/*A*_*Y*_)^1/*n*^ = 1.76 (2). In addition, Fig. 5 ▸ shows the ratio of the long- to short-axis intensities as a function of *Q* in the range 1 × 10^−3^ < *Q* < 1 × 10^−2^ Å^−1^ with a mean value of 7.11 (14), indicating a mean value for *a*/*b* = 1.80 (2). These results show that, on average, the pores have a larger dimension within the plane of bedding and a shorter dimension in the direction normal to the bedding.

We have also performed a measurement with the sample placed within a rotation device (Olsson *et al.*, 2013 ▸) which was originally designed to avoid sedimentation of colloidal particles. With the perpendicular-cut sample rotated uniformly around the incident neutron beam, the scattering intensity is an average over all azimuthal angles and is symmetric for given values of the scattering vector *Q*, *i.e.*

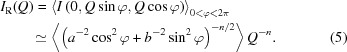
These data are given the designation R and the intensity *I*_R_(*Q*) = *A*_R_ *Q* ^−*n*^ ≃ *c^n^Q^−n^*, where 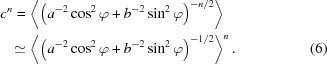
This generates an ellipse in polar coordinates, *c* ^2^ = (*a* ^−2^cos^2^φ + *b* ^−2^sin^2^φ)^−1^. The average of the azimuthal dependence is therefore given by 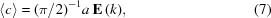
where *k* = (1 − *b* ^2^/*a* ^2^)^1/2^ is the eccentricity of the elliptical contours, and **E**(*k*) is the complete elliptical integral of the second kind (Abramowitz & Stegun, 1965 ▸; see also http://keisan.casio.com). With *a*/*b* = 1.76 (2) obtained from the fit, *k* = 0.823 (5) and 〈*c*〉/*a* = 0.799 (3). Then *A*_R_ = *A*_*Z*_ (〈*c*〉/*a*)^*n*^ = 6.76 (11) × 10^−5^ cm^−1^, compared with the experimental value of 7.11 (5) × 10^−5^ cm^−1^ from Table 1 ▸. Using the mean value for *a*/*b* = 1.80 (2) obtained directly from the ratio of the intensities in Fig. 5 ▸ gives *A*_R_ = 6.61 (6) × 10^−5^ cm^−1^.

**Figure 6:**
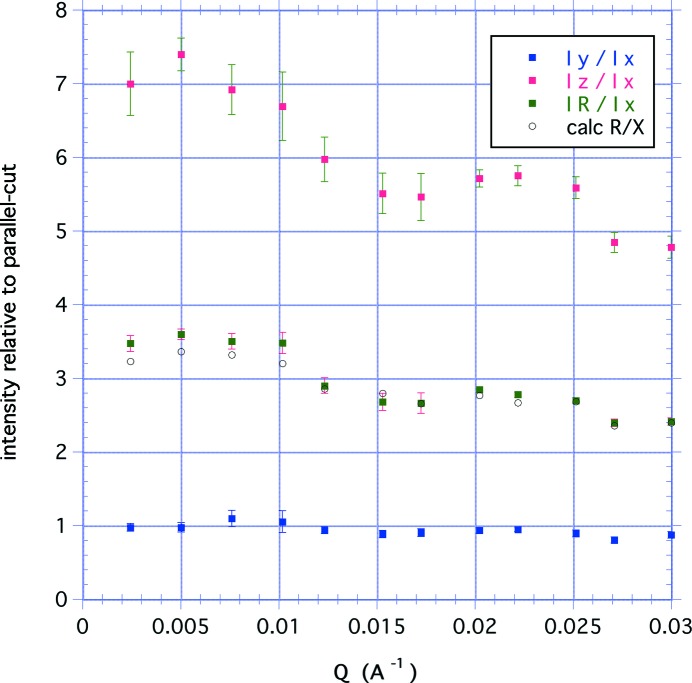
Values of the SANS intensity at selected values of the scattering vector for the three perpendicular-cut measurements, the short-axis *Y* curve, the long-axis *Z* curve and the rotated-sample R curve, normalized to the intensity of the parallel-cut *X* curve. Also shown is the intensity for the rotation calculated from the *Y* and *Z* curves.

The scattering intensities have been determined at various values of *Q* from the SANS data for each of the three data sets for the perpendicular-cut sample. These values, normalized to the intensity for the parallel-cut measurement, are shown in Fig. 6 ▸, indicating that the *Y* data for the perpendicular-cut sample are consistent with the *X* data for the parallel-cut sample. The figure also shows that the expected value for the rotation measurement calculated from the *Y* and *Z* data agrees well with the experimental results R.

## 4. USANS results   

The double-perfect-crystal USANS instrument measures the scattering for a given value of *Q* by summing the intensity in the vertical direction within the divergence ±*Q*_*m*_ of the incident beam of the instrument. That is, the smeared intensity *I*_sm,*X*_(*Q*) for the parallel-cut sample is a line average given by 
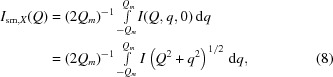
where *q* is the vertical component and *Q* the horizontal component of the scattering vector selected by the analyzer. The desmearing procedure is valid only for azimuthally symmetric scattering, and the smearing of the scattered intensity makes the analysis of USANS data problematic for asymmetric scattering.

**Figure 7:**
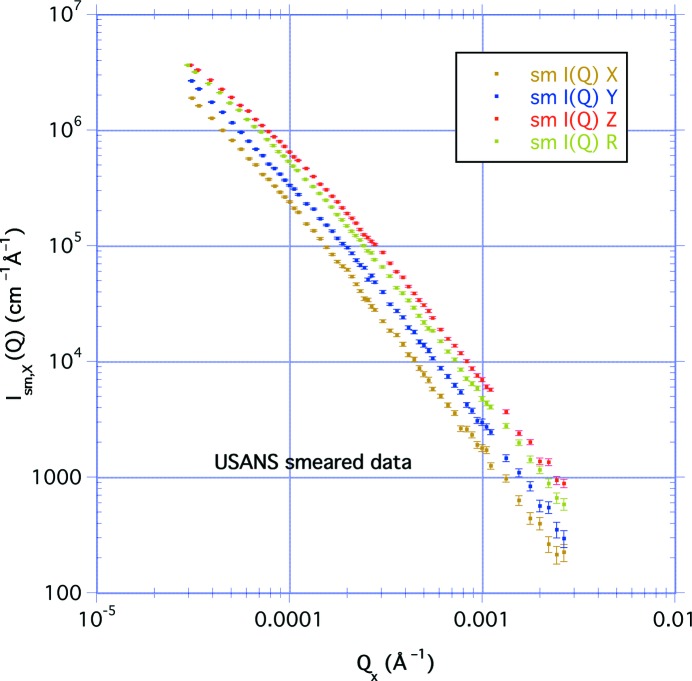
The slit-smeared USANS results plotted as a function of *Q*_*x*_, the scattering vector in the horizontal high-resolution direction. The *X* curve is for the parallel-cut sample, the *Y* and *Z* curves are for the perpendicular-cut sample with the bedding plane in the horizontal and vertical directions, respectively, and the R curve is for the rotation of the perpendicular-cut sample.

Fig. 7 ▸ shows the transmission-corrected slit-smeared USANS results as a function of *Q*_*x*_, the scattering vector in the horizontal high-resolution direction. For these measurements on the perpendicular-cut sample, *Y* designates that the bedding plane of the sample is placed in the horizontal plane of the instrument, resulting in elliptical contours on a two-dimensional detector with the longer axis in the vertical direction. Similarly, *Z* designates that the bedding plane is placed in the vertical plane of the instrument, with the longer axis of the elliptical contours in the horizontal direction.

In Appendix *A* [App appa] we compare isocontours for the two orientations of the perpendicular-cut sample with those for the parallel-cut sample in order to determine the expected relative intensities of the smeared USANS curves. The integral over the slit height is greater when the bedding plane is in the instrument horizontal direction, the *Y* measurement, than for the parallel-cut sample. This is what is observed in Fig. 7 ▸ for the smeared data, where the *Y* curve is no longer coincident with the *X* curve for the parallel measurement as was the case with the corresponding SANS measurements. Similarly, we find that the *Z* measurement with the bedding plane in the instrument vertical direction is reduced relative to the longer-axis SANS measurement. In addition, the curve for the perpendicular-cut sample rotated in the neutron beam also lies between the two, but is now closer to the *Z* curve.

We compare the four smeared USANS measurements in Fig. 7 ▸. We performed straight-line fits to the log–log plot, assuming that, over the range 2 × 10^−4^ < *Q* < 2 × 10^−3^ Å^−1^, the data are given by a power law *I*_s_(*Q*) = *AQ* ^−*m*^. The results are given in Table 1 ▸. Again, we find that, over this *Q* range, all the gradients are similar, with the exponent *m* = 2.16 (4). The large ratio (>5000) of the vertical to horizontal divergences means that the integral in equation (8) extends over a large range of *q* for all values of *Q*. Consequently, a power-law scattering such as *Q* ^−*n*^ becomes slit smeared as *Q* ^−*m*^, where *m* = *n* − 1 for *n* > 2. We find that this relationship holds as expected for the azimuthally symmetric measurements.

With the perpendicular-cut sample rotated uniformly around the incident neutron beam, the scattered intensity is an average of the smeared intensity over all azimuthal angles or orientations of the sample for given values of the horizontal component of the scattering vector. The rotation measurement is therefore given by 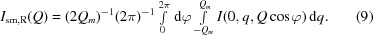
By analogy with equation (7) we obtain 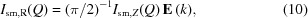
where *I*_sm,*Z*_(*Q*) is the smeared intensity when the bedding plane is in the vertical direction. Using the results given in Table 1 ▸, we find *A*_*Z*_/*A*_*Y*_ = 2.05 (2). With the exponent *m* = 2.16 (4), we find *a*/*b* = 1.39 (2), a value much lower than that determined from the SANS data. The value of *k* is 0.695 (10), and 〈*c*〉/*a* = (π/2)^−1^ **E**(*k*) = 0.865 (5). Then *A*_R_ = *A*_*Z*_(〈*c*〉/*a*)^*m*^ = 14.80 (21) × 10^−5^ cm^−1^, which compares reasonably with the experimental value of 15.76 (5) × 10^−5^ cm^−1^ from Table 1 ▸.

The curves deviate from a simple power law at the lower values of scattering vector. Straight line fits to *AQ* ^−*m*^ over the range 5 × 10^−5^ < *Q* < 2 × 10^−4^ Å^−1^ (a range much lower than in Table 1 ▸) give an average exponent *m* = 1.78 (9). The distinct separation in intensity for all four USANS plots for *Q* > 0.001 Å^−1^ becomes less evident at the lowest measured values of *Q* ≃ 3 × 10^−3^ Å^−1^ and the *Z* curve bends over more than the others. This might suggest that, at correlation distances much greater than 10 µm, the pores within the rock tend on average to become less anisotropic. This could also be an artifact of measurement close to the incident beam, and indeed some researchers (King *et al.*, 2015 ▸) discount data below *Q* = 1 × 10^−4^ Å^−1^ on account of multiple scattering caused by thicker samples than used here. Whatever the reason, this does not contradict the fact that the smaller scale pores are anisotropic, as shown by the SANS results.

**Figure 8:**
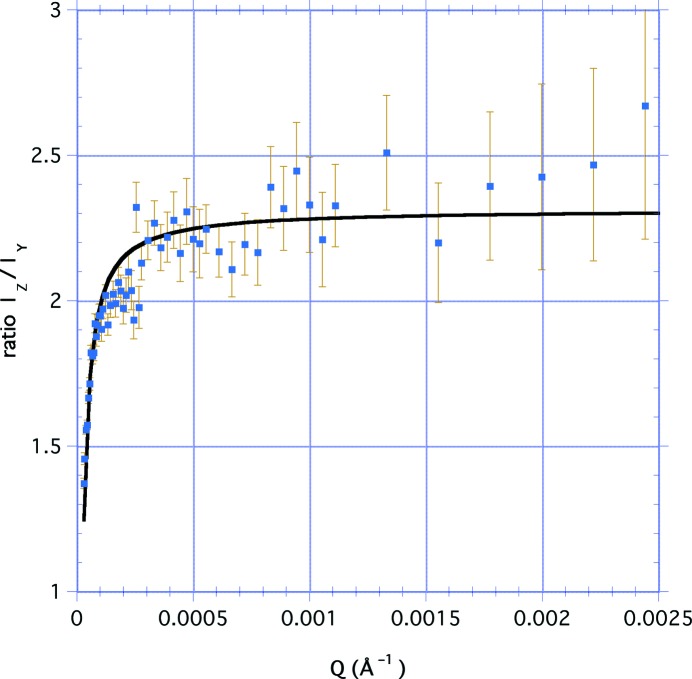
The ratio of the smeared USANS intensities for the perpendicular-cut sample with the bedding plane parallel and perpendicular to the scattering plane, plotted as a function of *Q* for the range 4 × 10^−5^ < *Q* < 2.5 × 10^−3^ Å^−1^. The line is a hyperbolic fit to the data: *I_Z_*/*I_Y_* = 2.31 (3) − 3.33 (26) × 10^−5^/*Q*.

The separation of the *Q* dependence from the azimuthal dependence can also be extended to the USANS data. Fig. 8 ▸ shows the ratio *I*_*Z*_/*I*_*Y*_ of the smeared intensity values for the perpendicular-cut sample with the bedding plane parallel and perpendicular to the scattering plane as a function of *Q* for the range 4 × 10^−5^ < *Q* < 2.5 × 10^−3^ Å^−1^. Clearly the model has broken down, but this might be expected since the ratio *a*/*b* must approach unity as the porosity becomes less anisotropic at values of *Q* well beyond the SANS range, corresponding to very large distances. The plot indicates that the ratio is a hyperbola that may be fitted to *I*_*Z*_/*I*_*Y*_ = *C* − *Q*_0_/*Q*, where *C* = 2.31 (3) and *Q*_0_ = 3.33 (26) × 10^−5^ Å^−1^. This suggests that there is little evidence of loss of anisotropy in the pores within this sample for a correlation distance at least of the order of 2π/*Q*_0_ ≃ 20 µm.

## 5. Desmearing USANS data   

We have desmeared the *X* and R data; the inversion procedure assumes knowledge of the smeared intensity over the entire range of *Q*, so the data are extrapolated to large *Q* to minimize truncation problems. We have performed straight-line fits to the log–log plot, assuming that, over the range 2 × 10^−4^ < *Q* < 2 × 10^−3^ Å^−1^, the data are given by a power law *I*(*Q*) = *AQ* ^−*n*^. Again we find that, over this *Q* range, the gradients are similar, with the exponent *n* = 3.15 (7). This is close to the value of *n* = 3.33 (4) obtained for the SANS data (see Table 1 ▸). Though it is not strictly valid we have also desmeared the *Y* and *Z* data.

**Figure 9:**
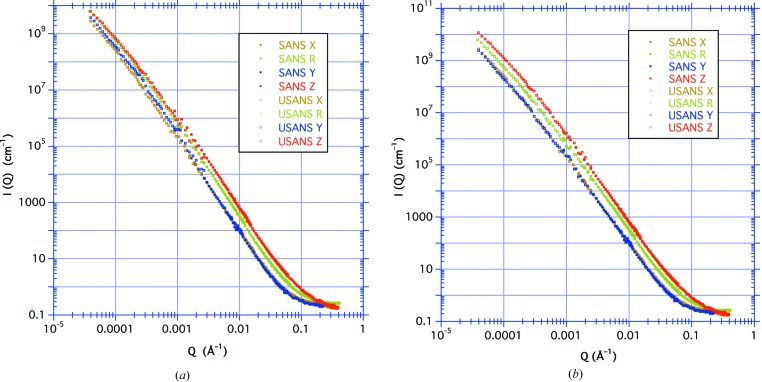
The combined results from the desmeared USANS (open square symbols) and the SANS (filled square symbols) measurements (*a*) before and (*b*) after modification to the USANS data. The parallel-cut (brown *X* curve) and the rotational (green R curve) measurements show a smooth transition, whereas for the perpendicular-cuts before modification the *Y* blue curve (with the bedding plane horizontal) has an upward jump at the transition and the *Z* red curve (with the bedding plane vertical) has a downward jump. After modification, the transitions are no longer so abrupt.

**Figure 10:**
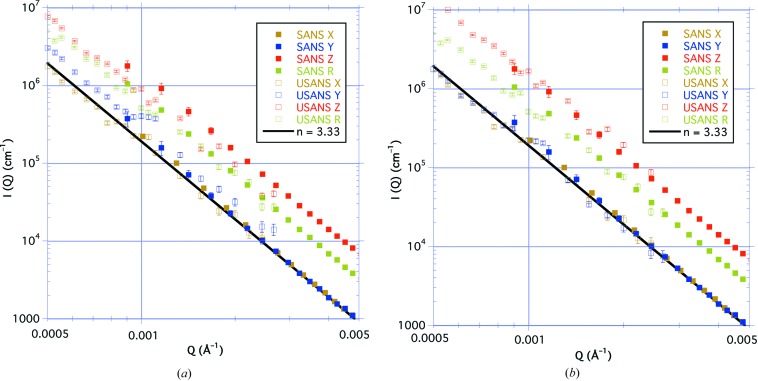
More detailed results from Fig. 9 ▸ in the vicinity of the overlap region of the SANS/USANS transition, showing (*a*) the upward jump for the *Y* curve and the downward jump for the *Z* curve before modification, and (*b*) the less abrupt transition after modification. The SANS data have filled square symbols and the USANS data have open square symbols. The line indicates the power law *AQ* ^−*n*^ for the *X* and *Y* curves, where *n* = 3.33.

Fig. 9 ▸(*a*) shows all four of the desmeared USANS results on the same plot as the SANS results. In the region around *Q* = 0.001 Å^–1^ where the SANS and USANS data meet, there is a smooth transition in scattering intensity for the symmetric parallel-cut case *X*, and also for the rotation case R for the perpendicular-cut sample which is quasi-symmetric. The desmearing algorithm correctly produces a curve for the azimuthally symmetric case that concatenates well with the SANS results. On the other hand, we observe a shift in the scattering intensity for both of the asymmetric perpendicular-cut measurements. These abrupt changes in the combined data can be better observed in Fig. 10 ▸(*a*), which shows the scattering intensity in the vicinity of the transition for both the *Y* and *Z* data at *Q* ≃ 0.001 Å^−1^.

We account for these changes in intensity at the crossover point as follows. The desmearing algorithm requires that the measured data be azimuthally symmetric, so that the scales of *Q* in the horizontal scan axis and vertical integration axis are commensurate, and the line averages are across the circular contours. This procedure determines a smooth transition from the SANS curve to the desmeared USANS result. The *Y* SANS curve is from a sector average at the lowest intensity of the two-dimensional data, whereas the *Y* USANS intensities are derived from line averages in the integration direction over contours more widely spaced than in the scan direction, resulting in an apparent greater intensity. Hence at the join of the two data sets there is a positive shift in intensity from one curve to the other. On the other hand, the *Z* SANS result is from a sector average at the highest intensity of the two-dimensional data. The *Z* USANS measurement is from a line average over contours more closely spaced than in the scan direction, resulting in an apparent lower intensity and a downward shift in the curve.

We have found a method by which the USANS data for the asymmetric scattering may be corrected for this desmearing distortion. The SANS data for the two cases are used to modify the USANS data for concatenation with the corresponding SANS data. The intensities *Y* and *Z* are multiplied by the appropriate factor such that the values for the horizontal and vertical components of *Q* are commensurate, the requirement for the desmearing algorithm. This procedure forces the spacing of the elliptical contours in each direction to become similar such that reciprocal-space steps in the integration axis have the same magnitude as those in the scan axis.

An elliptical contour has a corresponding circular contour of radius *r* = (*ab*)^1/2^, the geometric average of the two semi-axes of the elliptical contour. The ratio of the *Z* and *Y* prefactors for the SANS measurements shown in Table 1 ▸ gives the ratio *a*/*b* = 1.76 (2). For the *Y* measurement *b*/*r* = 0.75 (3), and for the *Z* measurement *a*/*r* = 1.33 (3). Artificially multiplying the *Y* intensity data by a factor (*b*/*r*)^*m*^ = 0.54 (5) and then performing the desmearing operation results in modified *Y* USANS data that concatenate with the *Y* SANS data and are consistent with the *X* USANS data. Similarly, multiplying the *Z* intensity data by a factor (*a*/*r*)^*m*^ = 1.84 (6) and then performing the desmearing operation results in modified *Z* USANS data that concatenate well with the *Z* SANS data. These results can be observed in Figs. 9 ▸(*b*) and 10 ▸(*b*). We have found similar results using other shale samples.

This procedure results in an intensity for asymmetric scattering that is consistent over the wide *Q* range offered by combined SANS and USANS. It requires the data themselves to correct for the line averaging over non-circular contours, with the aspect ratio *a*/*b* obtained from the SANS data. Confidence in this analysis can be obtained by considering the prefactors from the smeared USANS measurements given in Table 1 ▸. The ratio *A*_*X*_/*A*_*Y*_ = 0.609 (7) gives a value of *b*/*r* = (*A*_*X*_/*A*_*Y*_)^1/*m*^ = 0.79 (2), a value similar to that of 0.75 (3) obtained above from the *A*_*Z*_/*A*_*Y*_ SANS measurements. Moreover, the ratio of the modified USANS intensities for the *Z* and *Y* curves may be determined from the unmodified ratio shown in Fig. 8 ▸ by 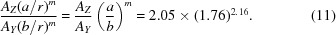
This gives a value of 6.95 (24) for the intensity ratio, compared with a value of 7.11 (14) for SANS shown in Fig. 5 ▸.

Note that modifying the intensity values by a given factor is in effect the same as altering the sample thickness by the same factor in the raw data correction. This begs a question regarding the accuracy of the sample thicknesses. It affects how well the data can be placed on an absolute basis, and indicates the importance of the correct sample thickness.

## 6. Summary   

We wish to modify USANS asymmetric data that follow a power law such that they concatenate well with data collected using pinhole SANS. The ratio of the intensities for the *Z* and *Y* SANS measurements depends on the aspect ratio *a*/*b* of the elliptical scattering contours and on the power law exponent *n*:
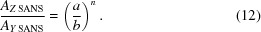
The two measured smeared USANS intensities may each be modified by the *a*/*b* ratio obtained from the SANS measurement to give 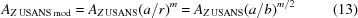
and 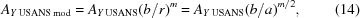
so that 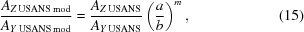
where *m* is the smeared USANS power-law exponent.

Ideally, if the power-law exponent were the same for both *Q* ranges, we would find the modified USANS intensity ratio to be the same as the SANS intensity ratio, *i.e.*

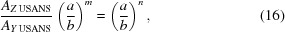
so that 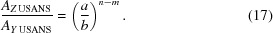
From Table 1 ▸ the SANS measurements give *A_Z_*/*A_Y_* = 6.63 (10), so that *a*/*b* = (*A_Z_*/*A_Y_*)^1/*n*^ = 1.77 (2). The value of *n* − *m* = 1.17 (6), so we might expect the USANS measurements to give *A_Z_*/*A_Y_* = 1.94 (7). For comparison, we find that the USANS measurements from Table 1 ▸ give *A_Z_*/*A_Y_* = 2.05 (2), so that *a*/*b* = (*A_Z_*/*A_Y_*)^1/(*n* − *m*)^ = 1.85 (6).

Alternatively, Fig. 4 ▸ indicates that similar results may be obtained by multiplying the variable *Q* by *b*/*r* = (*b*/*a*)^1/2^ for the *Y* USANS data, and by *a*/*r* = (*a*/*b*)^1/2^ for the *Z* USANS data.

## 7. Conclusions   

We have considered the analysis of the simplest form of azimuthal asymmetric scattering for measurements at scattering vectors below the normal SANS regime using a double-perfect-crystal USANS instrument. We would like to extract useful quantitative information from the measured data, for which no mathematical model of the scattering system is available. We have taken scattering measurements from thin shale rock samples that are known to have narrow pores that are slit like. These samples have been cut both parallel and perpendicular to the plane of bedding. The normal to the bedding plane is the unique axis that determines that the scattering contours for the perpendicular-cut samples are elliptical. The slit-smeared USANS results for the parallel- and perpendicular-cut samples in the two different orientations have been given as a function of scattering vector. The relative intensities of the various measurements have been qualitatively explained.

Quantitative statements regarding USANS data are less extractable because the desmearing algorithm is valid only for azimuthally symmetric scattering. This might be overcome if the elliptical contours were known to have the same aspect ratio for all measurable values of the scattering vector. We have developed a procedure that employs the ratio of the long and short axes of the elliptical contours observed in the SANS data. This result is used to modify the raw USANS intensity data, producing quasi-symmetric data that can be desmeared in the usual way. The final product is desmeared USANS data that can be successfully concatenated with the corresponding SANS data obtained in pinhole geometry.

A continuous rotation of the sample in the beam also produces quasi-symmetric data that can be desmeared. It is shown that this result agrees well with the results for the two orthogonal orientations of the perpendicular-cut sample. The reverse procedure – *i.e.* deducing the ratio of pore sizes parallel and perpendicular to the bedding from the rotational measurement – cannot be accomplished. We find that this particular sample has pores in the range 30 Å to 10 µm that have an aspect ratio of 1.7 (comparing dimensions in the bedding plane with those normal to the bedding), whereas we may consider the rock to be nearly isotropic for correlations over distances much greater than 30 µm.

## Figures and Tables

**Figure 11:**
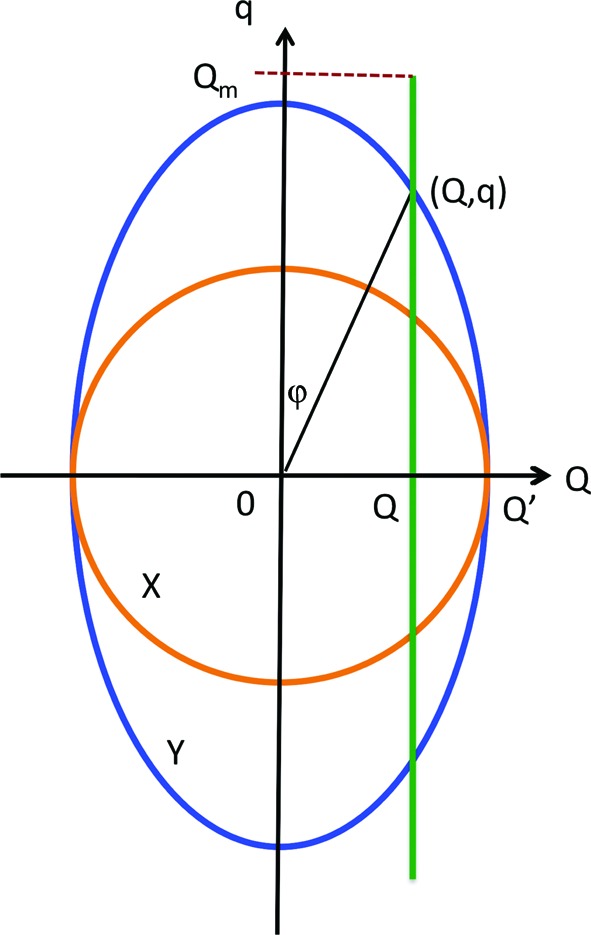
A schematic diagram in reciprocal space indicating isointensity contours of the same magnitude for the *X* (parallel-cut sample) measurement (circular contour, shown in orange) and for the *Y* (perpendicular-cut sample with the bedding plane horizontal) measurement (elliptical contour, shown in blue). The contours are tangential at the point (*Q*′, 0). The path of the slit-smearing integral at constant *Q* is shown in green and extends to ±*Q*_*m*_, corresponding to the maximum vertical divergence of the incident beam. The intensity at (*Q*, *q*) is greater for the *Y* measurement than for the *X* measurement for all *Q*, as is the integral given by equation (8).

**Table 1:** The straight-line fits of the SANS data to a simple power law *AQ* ^−*n*^ over the scattering vector magnitude range 1 × 10^−3^ < *Q* < 2 × 10^−2^ Å^−1^ with *n* = 3.33, together with the straight line fits of the smeared USANS data to a simple power law *AQ* ^−*m*^ over the scattering vector magnitude range 2 × 10^−4^ < *Q* < 2 × 10^−3^ Å^−1^ with *m* = 2.16

SANS measurement	Prefactor *A* (× 10^−5^ cm^−1^)	USANS measurement	Prefactor *A* (× 10^−4^ cm^−1^)
*X* curve, parallel-cut sample	2.21 (2)	*X* curve, parallel-cut sample	6.00 (5)
*Y* curve, short axis for perpendicular-cut sample	2.15 (3)	*Y* curve, bedding plane horizontal	9.86 (7)
*Z* curve, long axis for perpendicular-cut sample	14.26 (9)	*Z* curve, bedding plane vertical	20.25 (16)
R curve, rotation	7.11 (5)	R curve, rotation	15.76 (8)

## Appendix A

We assume that, over the range of measurements, the angular dependence of the scattering can be separated from the scattering vector dependence. The isointensity contours corresponding to the arrangement with the scattering plane normal to the bedding plane are elliptical in shape, given by equation (4), where φ is the azimuthal angle relative to the *Z* direction. That is, tanφ = *Q*/*q*, where *Q* and *q* are the horizontal and vertical components of the scattering vector, respectively. Fig. 11 ▸ shows two contours of the same intensity. The circular contour for the *X* measurement (with the parallel-cut sample) is tangential at the point (*Q*′, 0) with the elliptical contour for the *Y* measurement, with the long axis in the vertical direction and normal to the high-resolution scan. Since *a* > *b*, *I_Y_*(*Q*, *q*) > *I*_*X*_(*Q*, *q*) for all *q*. The smeared intensity *I*_sm_(*Q*) at a value of *Q* is given by equation (8). Hence, the relative smeared intensities are given by *I*_sm,*Y*_(*Q*, *q*) > *I*_sm,*X*_(*Q*, *q*).

Desmearing the USANS asymmetric data results in an upward shift in intensity observed in the *Y* direction for the perpendicular-cut sample relative to the *X* direction. (For the SANS pinhole measurements, the intensities in the *Y* direction are coincident with the *X* direction, the symmetric scattering case.) Similarly, there is a downward shift observed at the crossover point for the perpendicular-cut sample in the direction *Z* normal to the bedding plane. This is in contrast with both the *X* and R curves which concatenate smoothly.

Fig. 11 ▸ shows that the ratio of the contour spacings in the direction of the long axis is *a*/*r*, so that at (0, *q*), by analogy with Fig. 4 ▸, *I*_*Y*_/*I*_*X*_ = (*a*/*r*)*^m^*, where *r* is the radius of the corresponding circular contour. In order to make the elliptical contours commensurate for the desmearing algorithm, all intensities for the *Y* USANS curve must be multiplied by a factor (*r*/*a*)*^m^* = (*b*/*r*)*^m^*. Similarly, the intensities for the *Z* curve should be multiplied by (*a*/*r*)*^m^*.
